# Aortic sutureless bioprosthesis with concomitant Ferrazzi procedure, Coronary revascularization and Neo-fenestrated pericardial reconstruction: report of a case

**DOI:** 10.1186/1749-8090-10-S1-A309

**Published:** 2015-12-16

**Authors:** Faizus Sazzad, Vidanaptthirana Chirantha Puwalani, Chang Guohao, Julian Tan, Theodoros Kofidis

**Affiliations:** 1 Department of Cardiac Thoracic and Vascular Surgery National University Hospital, Singapore, Singapore; 2 Tan Tock Seng Hospital, Singapore, Singapore

## Background/Introduction

The clinical outcome of sutureless aortic bioprosthesis in concomitant complex heart surgery is still unclear.

## Aims/Objectives

We assessed a patient with large left ventricular aneurysm underwent concomitant procedure.

## Method

We report a case of 66 years Malay gentleman, diagnosed as large LV aneurysm (7.8 × 4.9 × 8.0 cm) secondary to previous anterior MI, Moderate aortic valve stenosis, Triple vessel coronary disease; underwent LV aneurysmectomy (Ferrazzi procedure) removal of LV thrombus, Sutureless aortic valve replacement (Perceval S), Coronary revascularization and Neo-fenestrated pericardial reconstruction with intra-aortic balloon counter pulsation support. At operation the large LV aneurysm was found at anterior-inferior apical area and was self-limiting leaking at the apical area (Almost ruptured) and was in-folded and got adherent to RV wall. Cardio-pulmonary bypass time was 199 min, and Cross-clamp 146 min.

## Results

Post operatively patient underwent resternotomy for bleeding and chest wash out on post-op day 1.Complicated with Bowel perforation and needed emergency exploratory laparotomy, Descending colon resection, with double barrel stoma creation, subsequently recovered well. Post op TTE showed LVIDD came down to 50 mm (Preoperative 72) with improved ejection fraction and good functioning sutureless valve.

## Discussion/Conclusion

Duration of surgery in complex concomitant procedure is an influencing factor for successful outcome. Sutureless aortic bioprosthesis reduces the operative length hence its use should be prioritized in such cases.

## Consent

Written informed consent was obtained from the patient for publication of this abstract and any accompanying images. A copy of the written consent is available for review by the Editor of this journal.

